# Poly-L-glutamate/glutamine synthesis in the cell wall of *Mycobacterium bovis* is regulated in response to nitrogen availability

**DOI:** 10.1186/1471-2180-13-226

**Published:** 2013-10-11

**Authors:** Deeksha Tripathi, Harish Chandra, Rakesh Bhatnagar

**Affiliations:** 1 Molecular Biology and Genetic Engineering Laboratory, School of Biotechnology, Jawaharlal Nehru University, New Delhi 110067, India; 2 Mailing address: School of Biotechnology, Jawaharlal Nehru University, New Delhi 110067, India

**Keywords:** *Mycobacterium bovis*, *Mycobacterium smegmatis*, Glutamine synthetase, Poly-L-glutamine/glutamate, Biofilm

## Abstract

**Background:**

The cell wall of pathogenic mycobacteria is known to possess poly-L-glutamine (PLG) layer. PLG synthesis has been directly linked to glutamine synthetase (GS) enzyme. *glnA1* gene encodes for GS enzyme in mycobacteria. PLG layer is absent in cell wall of avirulent *Mycobacterium smegmatis*, although *M. smegmatis* strain expressing GS enzyme of pathogenic mycobacteria can synthesize PLG layer in the cell wall. The role of GS enzyme has been extensively studied in *Mycobacterium tuberculosis*, however, little is known about GS enzyme in other mycobacterial species. *Mycobacterium bovis,* as an intracellular pathogen encounters nitrogen stress inside macrophages, thus it has developed nitrogen assimilatory pathways to survive in adverse conditions. We have investigated the expression and activity of *M. bovis* GS in response to nitrogen availability and effect on synthesis of PLG layer in the cell wall. *M. smegmatis* was used as a model to study the behaviour of *glnA1* locus of *M. bovis.*

**Results:**

We observed that GS expression and activity decreased significantly in high nitrogen grown conditions. In high nitrogen conditions, the amount of PLG in cell wall was drastically reduced (below detectable limits) as compared to low nitrogen condition in *M. bovis* and in *M. smegmatis* strain complemented with *M. bovis glnA1*. Additionally, biofilm formation by *M. smegmatis* strain complemented with *M. bovis glnA1* was increased than the wild type *M. smegmatis* strain.

**Conclusions:**

The physiological regulation of GS in *M. bovis* was found to be similar to that reported in other mycobacteria but this data revealed that PLG synthesis in the cell wall of pathogenic mycobacteria occurs only in nitrogen limiting conditions and on the contrary high nitrogen conditions inhibit PLG synthesis. This indicates that PLG synthesis may be a form of nitrogen assimilatory pathway during ammonium starvation in virulent mycobacteria. Also, we have found that *M. smegmatis* complemented with *M. bovis glnA1* was more efficient in biofilm formation than the wild type strain. This indicates that PLG layer favors biofilm formation. This study demonstrate that the nitrogen availability not only regulates GS expression and activity in *M. bovis* but also affects cell surface properties by modulating synthesis of PLG.

## Background

Tuberculosis remains one of the major causes of concern related to human health because of increasing incidence of mortality and morbidity all over the world. *Mycobacterium tuberculosis* and *Mycobacterium bovis* are the two pathogens, responsible for the disease in humans and animals respectively. The emergence of drug resistant strains of *M. tuberculosis* and failure of the current drug regimen has worsened the situation even more [1]. This has prompted renewed efforts to search for potential drug targets. In addition to this, there is an urgent requirement to bridge the massive gap in our understanding of pathogen’s complex biology to fight against disease.

Most of the studies on nitrogen metabolism have been focused primarily on other actinomycetes such as *Streptomyces* and *Coynebacterium* because of their role in industrial production of glutamine [2]. Nitrogen assimilatory pathways are very poorly understood in mycobacterial species, especially *M. bovis*. Studies related to nitrogen metabolism in pathogens may help in understanding of complex cellular mechanisms by which *M. bovis* survive in nitrogen stress inside the macrophages. Glutamine and glutamate are the two major amino acids that act as cellular nitrogen donors for synthesis of biomolecules inside the cell [3]. Hence, stringent regulatory pathways control the synthesis of glutamine and glutamate inside a bacterial cell [4]. In mycobacteria, assimilation of inorganic nitrogen and its conversion to glutamine and glutamate is carried out by glutamine synthetase (GS) and glutamate synthetase [5].

Virulent forms of mycobacteria secrete huge amounts of extracellular GS enzyme and are also known to possess poly-L-glutamine (PLG) layer in the cell wall. The PLG layer is absent in cell wall of saprophytic mycobacteria e.g*. M. smegmatis*. Earlier, the treatment of *M. tuberculosis* with an inhibitor of GS, L-methionine-S-sulfoxamine, or with antisense oligonucleotides to *glnA1* mRNA, has been shown to inhibit PLG formation in the cell wall [6,7]. It indicated indirect involvement of *glnA1* gene encoding the GS enzyme in the formation of PLG layer in *M. tuberculosis*. Later it was reported that expression of *M. bovis* GS in *M. smegmatis* resulted in synthesis of PLG layer in the cell wall and PLG significantly contribute strength to the cell wall against chemical and physical stresses such as lysozyme, SDS and sonication [8]. Because of its presence exclusively in the cell wall of virulent mycobacteria and its role in providing cell wall strength it would be interesting to study the factors that can affect PLG synthesis directly or indirectly.

**Figure 1:**
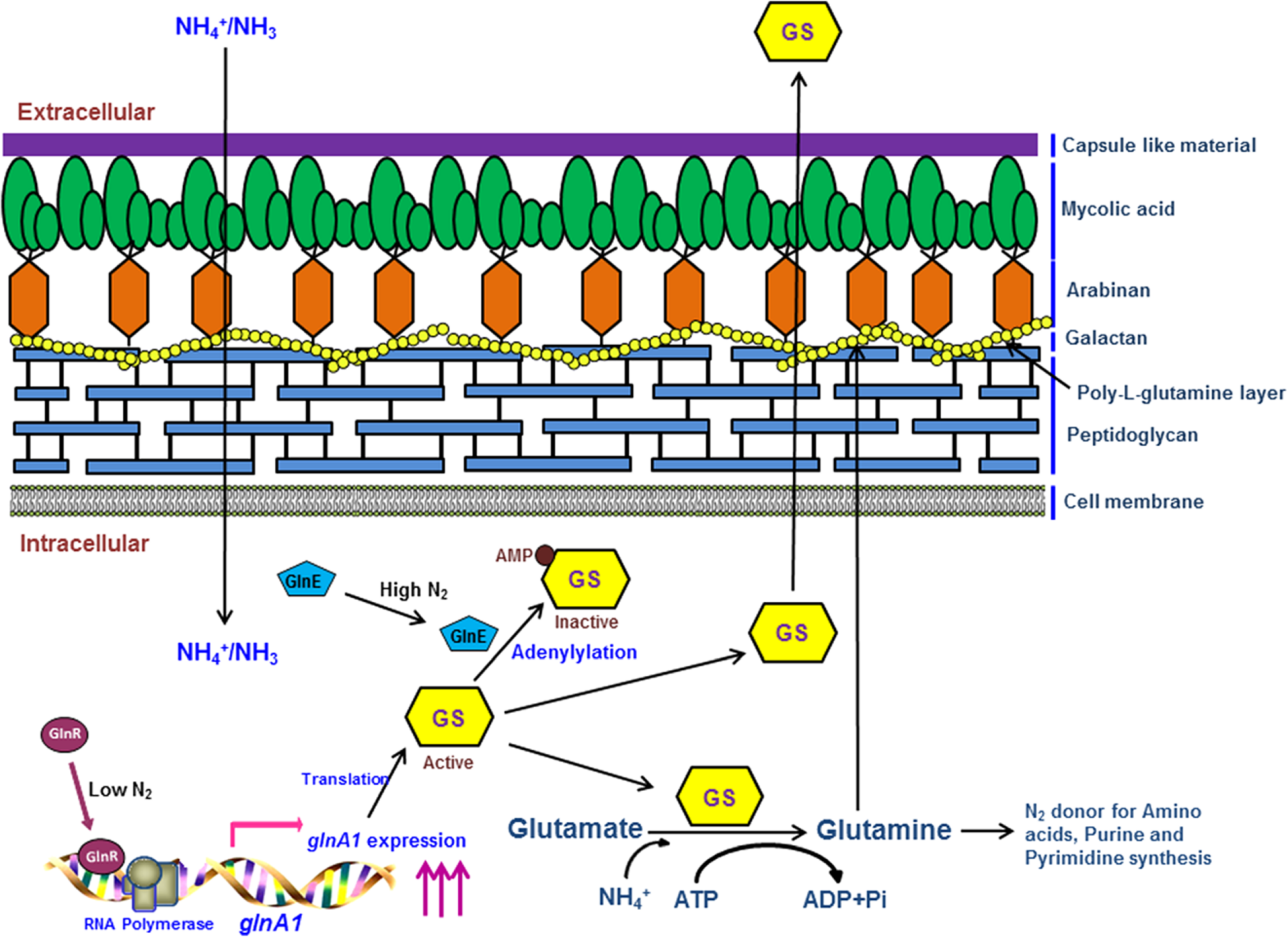
**Pictorial representation depicting role of glutamine synthetase in nitrogen metabolism and PLG synthesis.** In low nitrogen conditions GlnR acts as a positive regulator and activates transcription of glnA1 gene. In high nitrogen conditions GlnE acts as a negative regulator and adenylylated GS protein, which thus becomes inactive. GS, glutamine synthetase; ↑↑↑, up-regulation.

In view of the fact that formation of glutamine from glutamate and ammonia is a highly energy consuming process, *glnA1* gene is tightly regulated both at transcriptional and post translational levels in *M. tuberculosis*[9]. *M. bovis* and *M. tuberculosis glnA1* sequence exhibits 100% identity (both the coding DNA sequence and the upstream regulatory sequence). It has been previously reported that there are two promoters upstream to the *glnA1* gene in *M. tuberculosis*[10]. The size of transcript in low nitrogen condition was 1500 nucleotides while the same was around 1700 in high nitrogen conditions, so it was speculated that transcription starts from different promoters in different nitrogen conditions. In high nitrogen conditions the level of transcript is one fifth of the transcript level in low nitrogen conditions [10]. However, since then, effect of the two promoters when present independent of each other on *glnA1* expression in varying nitrogen concentrations has not been studied till date. Comparative analysis of the mRNA levels transcribed from the two promoters when they are present independent of each other, in response to varying nitrogen concentration, may reveal interesting information about gene expression in pathogenic mycobacteria. In low nitrogen conditions, GlnR protein acts as a positive regulator for *glnA1* gene in actinomycetes species [11]. It binds to upstream sequence of *glnA1* and activates transcription during nitrogen starvation (Figure 1). Furthermore, in high nitrogen conditions to evade the depletion of cellular glutamate levels due to conversion of all glutamate to glutamine the GS enzyme is modified post translationally [12]. In case of the nitrogen sufficiency, GlnE protein acts as a negative regulator and it adenylylates the GS enzyme at a conserved tyrosine residue at 406 position [13]. Hence, the adenylylated form of GS becomes inactive (Figure 1).

In this study, we investigated the behaviour of *glnA1* gene of *M. bovis* both at the mRNA and protein levels in response to nitrogen availability. The present study emphasizes on the effect of nitrogen concentration on expression levels of *glnA1* gene from the two different promoters when present independently or together. We have also studied the effect of nitrogen concentration on PLG layer synthesis in the cell wall of mycobacteria.

## Methods

### Bacterial strains and growth conditions

The bacterial strains and plasmids used in this study are listed in Table 1. *M. bovis* and *M. smegmatis* strains were routinely cultured in 7H9 broth (Difco) supplemented with 10% (v/v) albumin, dextrose and catalase (ADC), 0.2% (v/v) glycerol and 0.05% (v/v) Tween 80, at 37°C with shaking at 150 rpm. *Escherichia coli* DH5α (Novagen) was used for cloning experiments. *E. coli* DH5α was grown in Luria-Bertani medium. Kanamycin was used at concentration of 25 μg/ml for mycobacteria and 50 μg/ml for *E. coli* strains.

**Table 1:** Plasmids and strains used in this study

**Plasmids**	**Relevant characteristics**	**Source/Reference**
pGEM-T Easy	*amp*^*R*^*ori*^*pUC*^ (Cloning vector)	Promega
pMV261	*kan*^*R*^ (*Mycobacterial* shuttle non-integrative vector)	Stover *et al.,* 1991 [14]
pDS1	pGEM-T Easy containing *glnA1* coding sequence with native promoter	This work
pDS2	pMV261 containing *glnA1* coding sequence with native promoter	This work
pDS3	pGEM-T Easy containing *glnA1* coding sequence with P1 promoter	This work
pDS4	pMV261 containing *glnA1* coding sequence with P1 promoter	This work
pDS5	pMV261 containing *glnA1* coding sequence with P2 promoter	This work
**Strains**	**Relevant characteristics**	**Source/Reference**
DH5α	*supE44 ΔlacU(Φ80lacZΔM15) hsdR17 rec1 endA1 gyrA96 thi-1 relA1*	Novagen
*M. bovis* AN5	Wild Type	ATCC
*M. smegmatis* mc^2^	Wild Type	ATCC
MSFP	*M. smegmatis* containing pDS2	This work
MSP1	*M. smegmatis* containing pDS4	This work
MSP2	*M. smegmatis* containing pDS5	This work

Selection marker resistant to Ampicillin (*amp*^*R*^*)* and Kanamycin *(kan*^*R*^*).*

For creating low and high nitrogen conditions, mycobacterial strains were grown in 7H9 medium (without ADC enrichment) containing 3.8 mM ammonium sulphate and 60 mM ammonium sulphate respectively. It has previously been reported that a change in nitrogen concentration from 3 mM to 60 mM leads to a reduction in GS activity in wild type *M. smegmatis*[5].

The wild type *M. smegmatis* strain used in the study was complemented with only pMV261 vector and was used as a vector control. All work involving virulent strain was performed in Bio-safety Level-3 laboratory at Jawaharlal Nehru University, New Delhi.

### Cloning of *M. bovis glnA1* gene with its native promoter and construction of its deleted promoter variants in *M. smegmatis*

Cloning was performed using standard procedures. The *glnA1* gene with its upstream promoter region (1776 bp) was amplified using *M. bovis* genomic DNA as template. For PCR amplification of the gene, forward primer 1 with *Bam*HI site and reverse primer 2 with *Pst*I site (Additional file 1: Table S1), were used. The amplified DNA fragment was cloned in pGEM-T Easy PCR cloning vector, verified by sequencing and named as pDS1. The insert was excised from pDS1 by restriction digestion with *Bam*HI/*Pst*I, and then ligated in pMV261, *E. coli-Mycobacterium* shuttle vector, producing pDS2 plasmid. The resulting construct pDS2 was electroporated into wild type *M. smegmatis* strain and the transformed strain was named MSFP.

The *glnA1* promoter of *M. bovis* contains two regulatory promoters P1 and P2 (Figure 1). For the generation of construct carrying only the P1 promoter with *glnA1* gene downstream, the P2 promoter was deleted by direct PCR method. A forward primer 3 with *Bam*HI site immediately from the start of the P1 promoter and reverse primer 2 with *Pst*I site at the end of *glnA1* gene (Additional file 1: Table S1) were designed and were used to amplify *glnA1* gene which lacked the P2 promoter. The amplified (1561 bp) product was cloned in pGEM-T Easy vector (pDS3) and then sub-cloned in pMV261 vector at *Bam*H1-*Pst*1 sites (pDS4) (Table 1). Following this, for generation of construct carrying only P2 promoter with *glnA1* gene, P1 was deleted by the inverse PCR. In this method a primer was designed such that the sequence containing the P1 promoter was excluded. A forward primer 4 and reverse primer 5 were designed from the 3′ end of P1 promoter and 3′ end of P2 promoter respectively. PCR amplification by using template pDS2 resulted in the amplification of whole vector containing *glnA1* gene with P2 promoter (deletion of 31 bp) (Figure 1). The amplified PCR product was ligated after 5′ kinasing by T4 polynucleotide kinase and then the resulting construct was named as pDS5. The constructs pDS4 and pDS5 were then electroporated in wild type *M. smegmatis* and hence transformants obtained were named as MSP1 and MSP2 respectively.

### Growth patterns of recombinant *M. smegmatis* and *M. bovis* strains in low and high nitrogen conditions

Log phase cultures of *M. smegmatis* and *M. bovis* strains were inoculated in 7H9 medium containing low and high nitrogen conditions. The cultures were grown at 37°C at 200 rpm. The optical density was measured periodically at 600 nm.

### Semi quantitative RT-PCR and real time PCR

*M. smegmatis* and *M. bovis* strains were grown in low and high nitrogen conditions and total RNA was isolated by Trizol method. In brief, semi quantitative RT-PCR was performed using One Step RT-PCR Kit (Qiagen) according to manufacturer’s instructions. For *glnA1* gene, forward primer 10 and internal reverse primer 11 was used to amplify 400 bp fragment of the gene by using DNase I treated RNA as template. A *sigA* gene fragment was amplified using primers 8 and 12 as a loading control. The PCR conditions were, 50°C for 40 min, 94°C for 15 min and 24 cycles of 94°C denaturation for 30 sec, 58°C annealing for 30 sec and 72°C extension for 30 sec.

For real time PCR, DNase I treated RNA was taken for cDNA synthesis using High capacity cDNA reverse transcription kit (Applied Biosystems) employing random hexamer primers. The PCR reactions were run in ABI PRISM 7500HT sequence detection system (Applied Biosystems) using the following program: 95°C for 10 min and 40 cycles of 95°C for 10 sec, 60°C for 10 sec and 72°C for 10 sec. The forward primer 6 and reverse primer 7 were used for *glnA1* gene. The primer 8 and 9 were used for *sigA* gene and was used as internal control for data normalization. Each reaction was performed in triplicates. The relative changes in gene expression was calculated using the 2^-∆∆CT^ method and the data was represented in the form of fold change in gene expression, normalized to *sigA* gene and relative to the control condition.

### Determination of GS expression and activity

#### Extracellular activity

All strains were grown in low and high nitrogen conditions. The *M. smegmatis* strains were cultured for 2 days while *M. bovis* was cultured for 12 days. Then the culture filtrate was harvested. The culture filtrates were passed through 0.22 μm syringe filter and then concentrated 100 times of the original volume using 30 kDa molecular weight cut off Amicon filter (Millipore). The GS activity in the extracellular protein fraction was measured by γ-glutamyl transfer reaction as described previously [15] and was expressed as micromoles hydroxamate formed, based on a standard curve obtained with pure γ-glutamylhydroxamate purchased from sigma.

#### Intracellular activity

For the cytoplasmic protein fractions, cell pellets were taken and washed with 50 mM Tris–HCl pH 7.5 and digested with 10 μg/ml lysozyme. Cell pellets were resuspended in 1 ml of 50 mM Tris–HCl with 1X protease inhibitor. The *M. smegmatis* cell suspensions were sonicated on ice for 5–10 minutes while the *M. bovis* cell suspension was sonicated for 30 minutes, because the cell wall of virulent mycobacteria are relatively more resistant to physical stress like sonication. The GS activity in the cellular fraction was measured by the above mentioned protocol. The intracellular protein expression was determined by SDS-PAGE and western blotting by anti-GS antibody. The amount of total protein was measured by Bradford assay and equal amount of total protein was loaded for each sample.

### Isolation and estimation of PLG in mycobacterial strain

Cell pellet of exponential phase culture (200 ml) of all strains was harvested after growing in low and high nitrogen condition and cell wall was prepared. The PLG was purified as reported earlier [16]. The cell pellet was suspended in 10 ml of breaking buffer. The suspension was sonicated in an ice bath for 3–4 hrs.

The cell lysate was treated with 20 μl of 10 μg/ml ribonuclease and 20 units of deoxyribonuclease and kept overnight at 4°C.

Treated cell lysate was centrifuged at 27,000 g for 20 min, and the resulting cell wall-containing pellet was extracted with 2% (w/v) sodium dodecyl sulfate (SDS) for 2 h at 60°C to remove soluble protein and membrane. The extracted cell walls were washed extensively with PBS (phosphate buffer saline), distilled water and 80% (v/v) aqueous acetone to remove SDS.

Cell walls were suspended in a small volume of PBS and placed on a discontinuous sucrose gradient composed of 15, 25, 30, 40, and 60% (w/v) sucrose.

The gradient was centrifuged at 100,000 g for 2 hr.

The cell wall was settled at the 30 to 40% interface, whereas the associated PLG pelleted to the bottom of the tube.

The PLG material was transferred to a tube containing 80% Percoll (Sigma) in PBS-0.1% Tween 80 and centrifuged at 100,000 g for 20 min.

This allowed formation of a gradient *in situ* and distinct banding of the insoluble, pure PLG. The presence of PLG was confirmed by GC-MS analysis, after hydrolysis of the samples at 110°C for 20 h with 6 N HCl followed by esterification with heptafluorobutyryl isobutyl anhydride [17]. GC-MS was done at Advanced Instrumentation Research Facility, JNU New Delhi by Shimadzu GC-MS 2010, and Rtx-5 MS capillary column (Restek) with an oven temperature range of 90-180°C (5 min) at 4°C/min raised to 300°C at 4°C/min. The injection temperature used was 280°C along with an interface temperature of 290°C. MS data were analyzed in the NIST05.LIB and WILEY8.LIB chemical libraries.

### Immunogold localization of PLG by transmission electron microscopy

Immunoelectron microscopy was performed to confirm the presence of PLG in the cell wall of *M. smegmatis* and *M. bovis* strains grown under different nitrogen conditions. Immunogold localization was done as described earlier [18] at the Transmission Electron Microscopy Facility, Advanced Instrumentation Research Facility, JNU, New Delhi. Briefly*,* cells from log-phase cultures of *M. bovis* and *M. smegmatis* strains were harvested and washed with 0.1 M phosphate buffer. The cells were treated with immune gold fixative (4% paraformaldehyde and 0.5% glutaraldehyde in 0.1 M phosphate buffer), then washed and embedded in 2.5% agar. The agar-encased bacteria were then dehydrated and embedded using LR white resin (Electron Microscopy Sciences). Thin sections (100 nm) were obtained using Leica Ultracut (Leica, Germany) and collected on Nickel grids (200 mesh; Electron Microscopy Sciences). For localization, monoclonal anti-PLG antibody (1:100) (Sigma) was used. The grids were washed and subsequently treated with gold (10 nm) conjugated - anti mouse IgG. Mice pre-immune serum was used as a negative control. The immunolabeled sections were stained with uranyl acetate and viewed using a Jeol 2100 F transmission electron microscope (Jeol Analytic Instruments) at an acceleration voltage of 120 KV.

### Biofilm formation

Biofilm formation was observed by growing static cultures of mycobacteria without shaking in 7H9 medium without Tween 80 at 37°C. Biofilm formation was assayed by crystal violet staining method developed by Reicht *et al.*[19,20]. Briefly, 200 μl of stationary phase cultures (A_600_ normalized to 1) were added to 7H9 medium in polystyrene culture plates for biofilm formation and in culture tubes for pellicle formation. After incubation of static culture of *M. smegmatis* strains for 2 days and *M. bovis* for 2–3 weeks, biofilm was quantified by removing the medium carefully and staining with 1% crystal violet for 45 min. The wells were washed three times with water and air-dried. The dye was solubilized with 80% ethanol and A_550_ was measured.

## Results

### Generation of *glnA1* promoter variants

**Figure 2:**
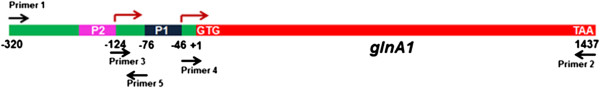
**Schematic representation of *glnA1 *promoter.***glnA1* gene with two promoters P1 and P2. +1 represents *glnA1* translational start site. The red arrow represents the transcriptional start site. The black arrow represents the position of primers used to make deletion variants of the *glnA1* promoter.

Figure 2 shows a schematic representation of the deletion variants of the promoter. *M. bovis* contains two native promoters P1 and P2 within 320 bp upstream of *glnA1* gene (start codon designated as +1). 124 bp upstream of *glnA1* start codon was taken as P1 promoter. Further, from 320 bp upstream sequence, 31 bp (-46 to -76) was deleted from the native promoter and taken as P2 promoter. The native, P1 and P2 promoter with *glnA1* gene were used for further characterization in response to nitrogen limitation and excess.

### Growth characteristics

**Figure 3:**
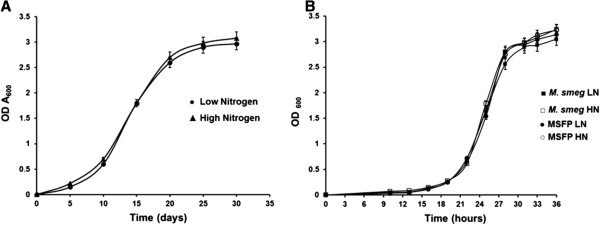
**Growth of the mycobacterial strains in low and high nitrogen broth culture. A**. OD_600_ of wild type *M. bovis* was inoculated to an initial optical density of 0.006 - 0.008 in 7H9 medium containing (●) low nitrogen (3.8 mM ammonium sulphate) and (▲) high nitrogen (60 mM ammonium sulphate). **B**. OD_600_ of wild type *M. smegmatis* and MSFP in low and high nitrogen broth culture. Wild type *M. smegmatis,* low nitrogen (■), high nitrogen (□); MSFP, low nitrogen (●), high nitrogen (○). Data is mean ± SD of values obtained from three independent cultures. LN, low nitrogen; HN, high nitrogen.

*M. bovis* strain was grown in low and high nitrogen medium and growth profile was studied by measuring optical density at 600 nm. No significant difference was observed in the growth of *M. bovis* when cultured in low nitrogen medium as compared to growth in high nitrogen medium (Figure 3A). This indicated that *M. bovis* was able to acquire nitrogen from other sources in the medium (L-glutamic acid, ferric ammonium citrate and ammonium sulphate). Same was the case when growth of wild type *M. smegmatis* and MSFP was studied in low and high nitrogen conditions (Figure 3B). Comparable growth profile was observed for all the strains in both low and high nitrogen conditions, clearly indicating that low nitrogen conditions doesn’t hamper the growth of the strains.

### Relative quantification of *glnA1* transcript of recombinant *M. smegmatis* strains

**Figure 4:**
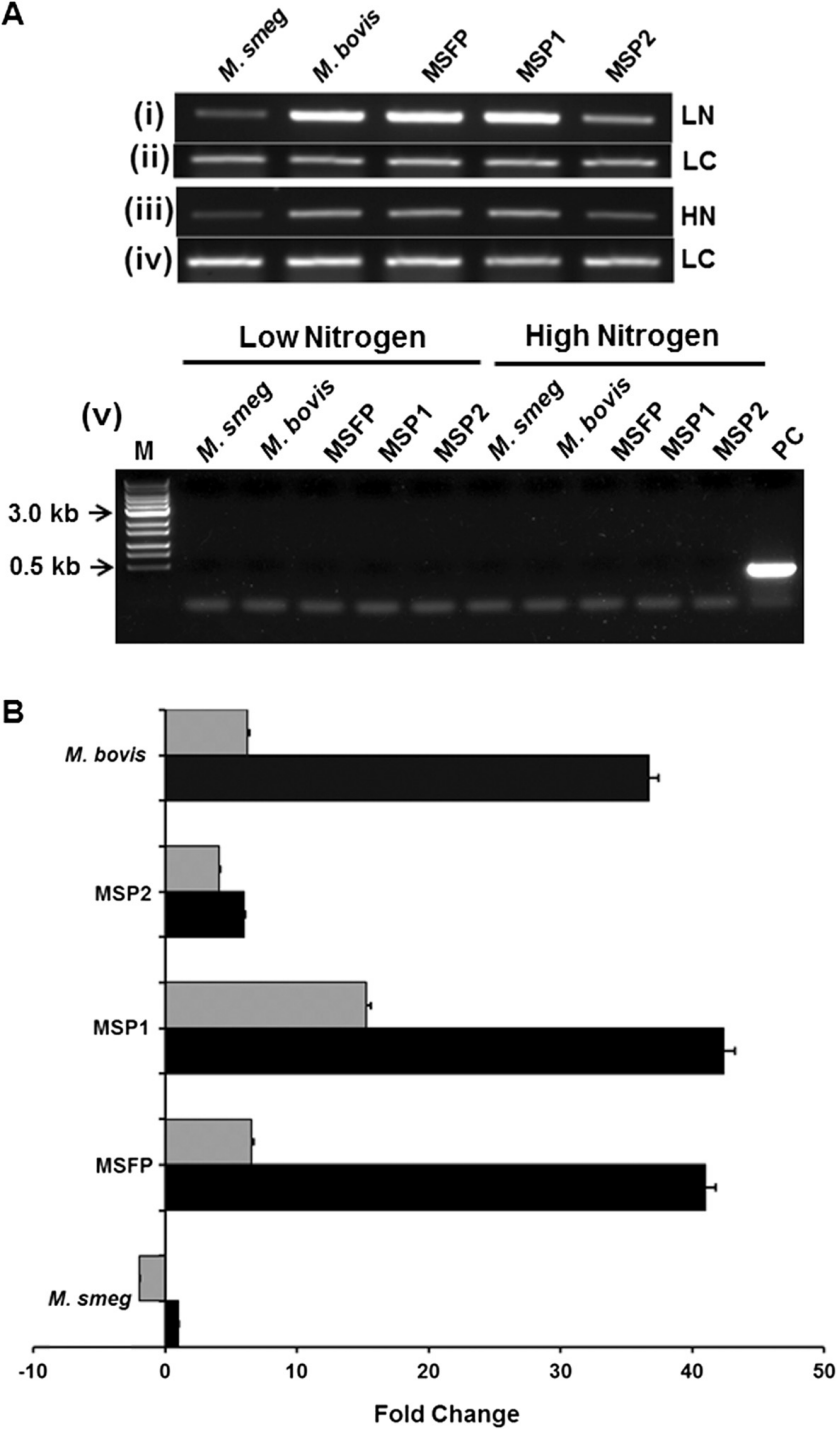
**Analysis of *glnA1 *transcription in mycobacterial strains in low and high nitrogen condition. A**. For semi-quantitative reverse transcriptase PCR analysis, mycobacterial strains were grown in low and high nitrogen condition. *glnA1* transcripts in (i) low nitrogen and (iii) high nitrogen condition. *sigA* loading control of respective test samples in low nitrogen (ii) and (iv) high nitrogen condition. (v) Genomic DNA contamination PCR analysis by *sigA* amplification without reverse transcriptase of respective test samples grown in low and high nitrogen condition. Lane M, marker; lane PC, positive control. **B**. For real-time (qRT-PCR) analysis, the expression profiles of *glnA1* gene in low nitrogen (black bars) and high nitrogen (grey bars) conditions were compared with respect to their corresponding *M. smegmatis* wild-type strain in low nitrogen. Data shown are linear fold change normalized to *sigA* expression level. The transcripts were quantified by a SYBR Green-based real-time PCR assay as described under “Materials and Methods.” The experiments were repeated three times, and data from one of the representative experiments are presented. LN, low nitrogen; HN, high nitrogen; LC, loading control.

Semi-quantitative RT-PCR assays were performed with RNA obtained from different strains grown in low and high nitrogen condition. *M. smegmatis* strain (MSFP and MSP1) showed up-regulation of *glnA1* transcript in low nitrogen as compared to high nitrogen condition. The *glnA1* transcript of *M. bovis* was also higher in low nitrogen than in high nitrogen condition, while MSP2 had no effect on *glnA1* mRNA level in different nitrogen conditions (Figure 4A, panel i and iii).

Real time PCR was performed further to study *glnA1* expression quantitatively in low and high nitrogen conditions for MSFP, MSP1, MSP2, wild type *M. smegmatis* and *M. bovis* strains. The *glnA1* expression levels in wild type *M. smegmatis* in low nitrogen condition was taken as the reference point in order to calculate the fold change in recombinant strains. The data obtained from real time PCR was normalized to *sigA* expression levels, as an internal control. It was observed that in case of nitrogen starvation, the expression of *glnA1* gene in MSFP and MSP1 strains was highly up-regulated. It was observed that in MSFP *glnA1* expression was ~ 40 fold high in ammonium starvation, while it was just ~ 6 fold more in high nitrogen conditions as compared to wild type *M. smegmatis* (which was taken as a reference point to calculate fold change for all the strains) (Figure 4B). In MSP1 *glnA1* expression in low and high nitrogen conditions was up-regulated ~ 42 and ~ 15 fold respectively. The *glnA1* expression in MSFP in high nitrogen was ~ 6 fold less than expression in low nitrogen while the same was only ~ 3 fold in MSP1. In case of MSP2, the expression of *glnA1* gene was comparable in both low and high nitrogen conditions. In case of *M. bovis*, the expression of *glnA1* was also ~ 36 fold up-regulated in low nitrogen conditions as compared to ~ 6.2 fold in high nitrogen conditions. Hence it was observed that in the strains, MSFP and *M. bovis*, where both the promoters P1 and P2 were present upstream to *glnA1*, the difference in the gene expression levels in low and high nitrogen conditions were significantly higher as compared to the difference in expression levels in strains having single promoter. It was concluded that deletion of any one of the two promoters decreased the stringent regulation of *glnA1* gene at the transcriptional level.

### GS specific activity and expression in response to nitrogen limitation and excess

**Figure 5:**
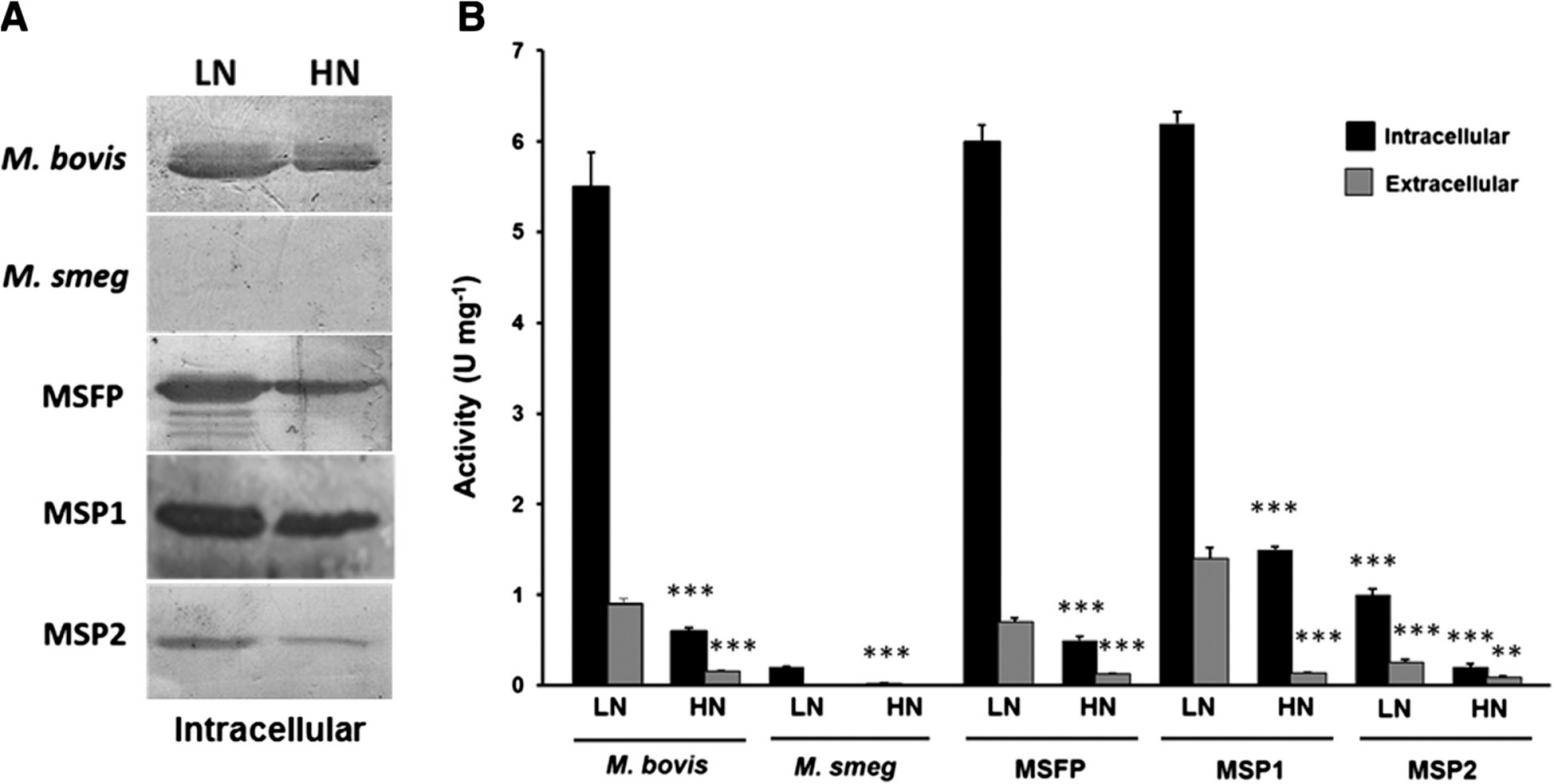
**GlnA1 enzyme activity and protein expression of mycobacterial strains in low nitrogen and high nitrogen medium. A**. Enzyme activity in the intracellular fractions (black bars) and extracellular fractions (grey bars). ***, ** The p-value given show the statistical significant of the change in GS specific activity between low nitrogen to high nitrogen. P < 0.001 for *** and P < 0.01 for ** was regarded as a statistically significant change in specific activity from low nitrogen to high nitrogen. **B**. Western blots of the intracellular fractions analyzed by the anti-GS antibodies. LN, low nitrogen; HN, high nitrogen.

Response to nitrogen availability for GS enzyme was studied by measuring cellular GS activity by γ-glutamyl transferase assay [15]. Exponential phase culture of MSFP, MSP1, MSP2, wild type *M. smegmatis* and *M. bovis* was harvested and cell pellet of 10 ml culture was further used for determining intracellular GS activity. Upon exposure to the nitrogen limiting conditions, the cellular GS activity in *M. bovis*, MSFP, MSP1 and MSP2 was 9.16, 12, 4.4 and 5 times higher than the high nitrogen condition respectively. Intracellular GS activity for all strains grown in high nitrogen condition was much less as compared to the activity in low nitrogen conditions (Figure 5B). Intracellular GS specific activity in MSP2 strain was 1 U/mg in low nitrogen and 0.2 U/mg in high nitrogen condition which was much less as compared to GS activity in MSFP and MSP1 strain. The GS activity in extracellular fraction followed the same trend in all strains (Figure 5B). Western blotting of the intracellular protein fraction was done by using anti-GS antibodies (Figure 5A). It was observed that in all strains the GS expression was higher in low nitrogen condition than high nitrogen condition. Although it was observed from western blotting result that the amount of GS in low nitrogen condition of MSP2 was very less but the activity of the enzyme was relatively higher than the activity of the enzyme in high nitrogen conditions of all the strains. This is in accordance with earlier findings that in high nitrogen conditions GlnE protein adenylylates the GS protein at a conserved tyrosine residue and hence, the enzyme becomes inactive.

### Estimation of PLG from *M. bovis* and recombinant *M. smegmatis* strains

Effect on cell wall PLG in response to nitrogen availability was studied by isolation and estimation of PLG layer. For this the strains were grown in low and high nitrogen conditions and then the cell wall was isolated. It was observed that no pellet settled in the sucrose gradient when *M. bovis*, MSFP, MSP1 and MSP2 strains were grown in high nitrogen medium (Table 2). Hence it was concluded that the PLG content in the cell wall was drastically reduced (below detectable limits) when *M. smegmatis* and *M. bovis* strains were grown in high nitrogen medium. In high nitrogen conditions, most of the GS enzyme inside the cell is in adenylylated state [21] and thus it may become inactive and unable to form PLG layer. Although in case of limiting nitrogen conditions, PLG was obtained from the cell wall of *M. bovis*, MSFP, MSP1 and MSP2 strains. For wild type *M. smegmatis,* no PLG was obtained from the cell wall in both low and high nitrogen conditions, as expected. GC mass analysis of the purified material confirmed the presence of PLG (data not shown).

**Table 2:** Estimation of PLG

**Strain**	***M. bovis *(gm)**		***M. smeg *(gm)**		**MSFP (gm)**		**MSP1 (gm)**		**MSP2 (gm)**	
	**LN**	**HN**	**LN**	**HN**	**LN**	**HN**	**LN**	**HN**	**LN**	**HN**
**Dry cell weight**	2.78±0.3	2.85±0.2	3.04±0.4	3.3±0.19	3.876±0.16	3.34±0.18	2.98±0.24	3.008±0.11	3.43±0.14	3.07±0.25
**Cell wall weight after sonication**	1.08±0.2	1.34±0.1	1.24±0.15	1.43±0.23	1.87±0.11	1.56±0.12	1.32±0.32	1.47±0.07	1.36±0.11	1.57±0.11
**Insoluble cell wall after SDS extraction and acetone wash**	0.870±0.1	0.680±0.08	0.768±0.08	0.567±0.13	1.02±0.2	0.98±0.14	0.69±0.09	0.75±0.08	0.62±0.07	0.73±0.12
**Poly-L-glutamine pelleted after sucrose gradient centrifugation**	0.070±0.03	No Pellet	No Pellet	No Pellet	0.087±0.017	No Pellet	0.078±0.011	No Pellet	0.056±0.02	No Pellet
**Poly-L-glutamine purified after percoll run**	0.069±0.02	No PLG	No PLG	No PLG	0.075±0.012	No PLG	0.056±0.02	No PLG	0.034±0.01	No PLG

Data are mean ± SD of triplicate sample and are representative of three independent experiments.

### Immunogold localization of PLG by transmission electron microscopy

**Figure 6:**
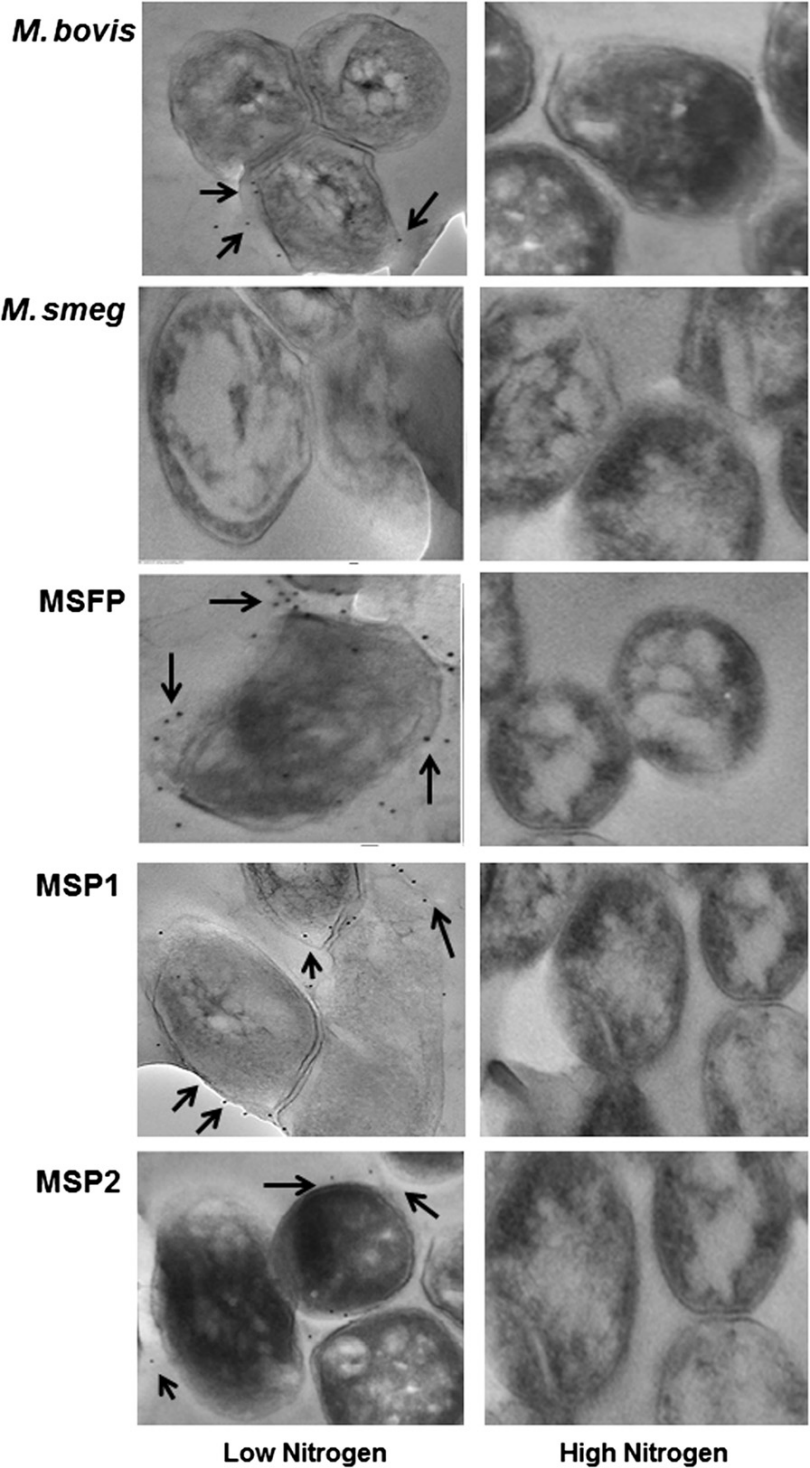
**Immunogold localization of PLG in the cell wall of mycobacteria during nitrogen availability.** Shown are transmission electron micrographs of the wild type *M. bovis* and recombinant *M. smegmatis* strain (MSFP, MSP1 and MSP2) in low and high nitrogen condition. The black arrows shown in the images marked the gold particle around the cell wall periphery in low nitrogen condition.

In order to validate the above observation, immunogold localization study was done to localize PLG in the cell wall. Exponential phase cultures were taken for localization studies. Gold particles were observed at the cell periphery of bacteria gown in nitrogen limiting conditions (Figure 6). While no gold particles were seen at the cell periphery of mycobacterial strains grown in high nitrogen condition, as expected. Interestingly the less number of gold particles was found in the MSP2 strain as low amount of GS expression and PLG formation.

### Effect on biofilm formation

**Figure 7:**
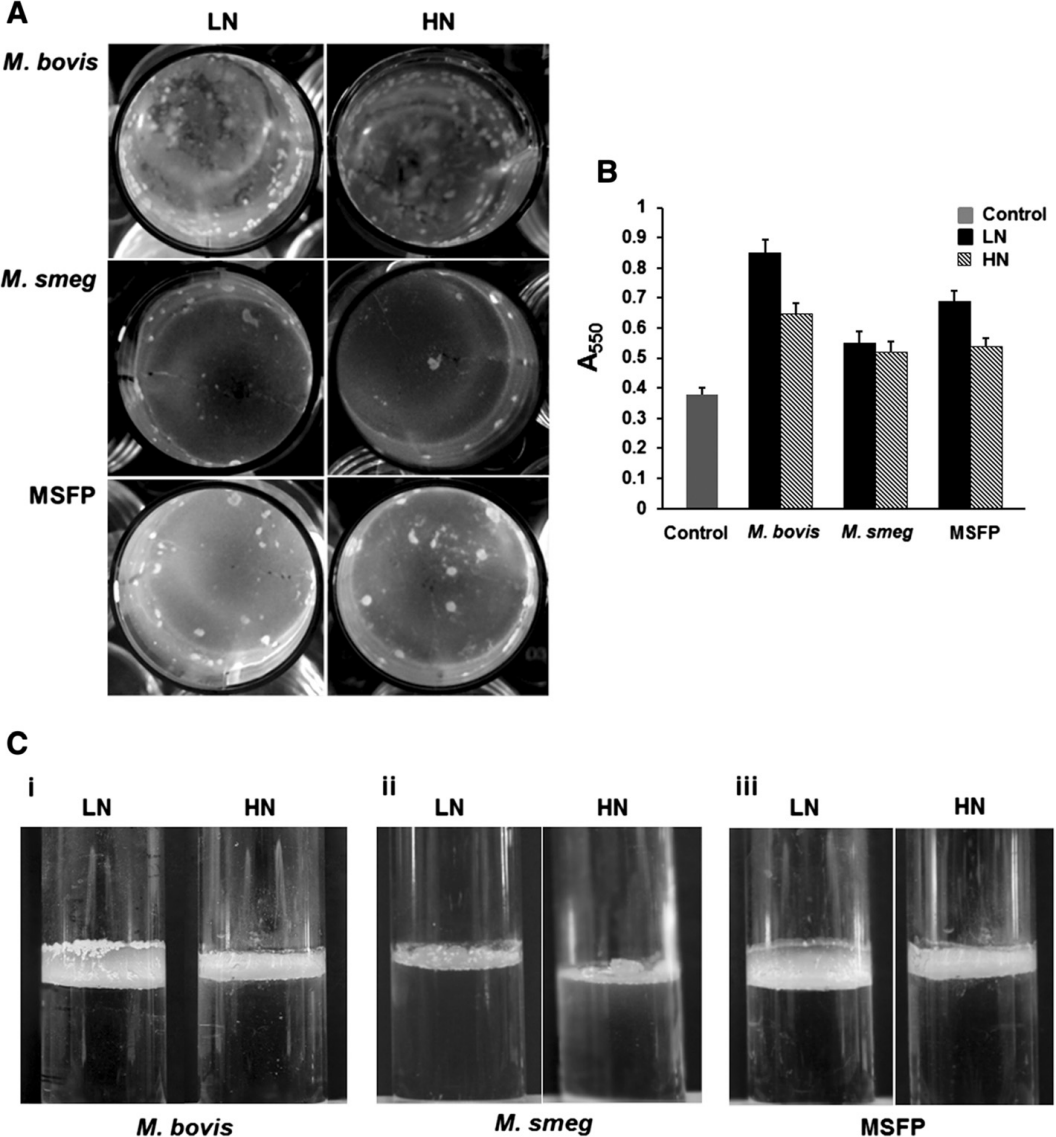
**Biofilm and pellicle formation under low and high nitrogen condition. A**. *M. bovis,* wild type *M. smegmatis* and MSFP were grown 7H9 medium to form biofilm in low and high nitrogen medium. **B**. Biofilm formation assayed using the 1% crystal violet (CV) staining assay. Cells in low nitrogen (black bars), High nitrogen (crossed bars) and control (grey bars) in 7H9 media were grown in low and high nitrogen on polystyrene plates. The experiments were repeated three times with similar result. Control, medium only. **C**. Pellicle formation at the air-liquid interface of the standing 7H9 culture by strains *M. bovis* (i)*, M. smegmatis* (ii) and MSFP (iii) in low and high nitrogen condition. Results are representative of at least three independent experiments. LN, low nitrogen; HN, high nitrogen.

It was earlier reported that a ∆*glnA1* strain of *M. bovis* that lack PLG layer in the cell wall was found to be defective in biofilm formation [8]. Our studies on biofilm formation were found to be in accordance with earlier reports. MSFP and *M. bovis* strains were defective in forming biofilm in high nitrogen on a polystyrene surface. Both strains showed ~ 25% reduction in biofilm formation in high nitrogen condition as compared to low nitrogen condition while *M. smegmatis* strain showed no difference in the biofilm formation (Figure 7A and B). The pellicle formation for the MSFP and *M. bovis* strains were also significantly less in high nitrogen as compared to the low nitrogen condition (Figure 7C). Interestingly, the pellicle formation by *M. smegmatis* strain complemented with *M. bovis glnA1* was enhanced than the wild type. It reiterates the involvement of *glnA1* in modulating the cell surface properties of mycobacteria [8].

## Discussion

Nitrogen metabolism has been studied in detail in industrially important organisms such as Streptomyces and Corynebacteria but there have been very few reports on nitrogen metabolism of mycobacterial species. Earlier, several studies have reported that *glnA1* gene is up-regulated in nitrogen starvation in *M. tuberculosis* and *M. smegmatis*[5,12] but this study emphasizes on behaviour of *glnA1* locus of *M. bovis* at both transcriptional and translational levels by altering nitrogen concentration in the medium. Also nitrogen conditions modulate the cell wall properties by altering synthesis of PLG layer in mycobacteria.

The conversion of glutamate to glutamine demands high energy consumption inside the cell. Because of this reason the expression of *glnA1* gene is tightly regulated in most mycobacterial species. The transcription of *glnA1* gene is regulated in *M. tuberculosis* by dual promoters [10]. The P1 promoter, present just upstream to *glnA1* gene is low nitrogen responding promoter while the P2 promoter, upstream to P1 is high nitrogen responding promoter [10]. Further regulation is driven by GlnR protein which has putative binding site in the P1 promoter. GlnR binds to the P1 promoter and activates transcription during nitrogen starvation [11]. In this study, we have studied the expression level of *glnA1* gene of *M. bovis* in response to nitrogen availability, when the two promoters P1 and P2, are present independently or together. The real time data observed are in accordance with the earlier findings about the regulation of *glnA1* gene at transcription level in response to nitrogen availability [11,12]. The results clearly showed up-regulation of *glnA1* expression in *M. bovis* and MSFP strains in low nitrogen conditions as compared to high nitrogen conditions. MSFP, MSP1 and *M. bovis* strains have P1 promoter upstream to the *glnA1* gene and P1 promoter has binding site for GlnR protein. GlnR binds to the P1 promoter and activates transcription in low nitrogen conditions [11]. This may be the reason for the differences observed in the expression level of the gene in low nitrogen and high nitrogen conditions in these strains. While, on the other hand in MSP2 strain there was no difference in *glnA1* expression level in low and high nitrogen conditions. This may be due to lack of P1 promoter and hence GlnR binding site. Also, it can be observed that the difference in gene expression in low and high nitrogen conditions are higher in MSFP and *M. bovis* strains that have both the promoters upstream to the *glnA1* gene. This difference is somewhat reduced in MSP1 and completely lost in MSP2 strain. It has been reported earlier that P1 promoter in *M. tuberculosis* is σ ^60^ type promoter [10]. σ ^60^ is expressed in nitrogen limiting conditions, it recognizes the P1 promoter and transcription starts from P1 promoter.

In addition to regulation at the transcriptional level, GS enzyme encounters a second regulation at post translational level. GlnE protein adenylylate the GS protein in high nitrogen condition and thus makes it inactive [13,22]. In all the strains, the difference in GS activity in ammonium starvation to ammonium pulse was significantly higher than the difference in expression at mRNA level. Hence, this marked difference observed in GS activity with change in nitrogen conditions in *M. bovis*, MSFP and MSP1 may be because of two possible reasons. First, there is a stringent regulatory mechanism exhibited by GlnR protein at the transcriptional level because of which the transcript of *glnA1* gene itself, is significantly low in high nitrogen conditions. Secondly, after translation, GlnE protein comes into play and modifies the GS enzyme in high nitrogen conditions which makes GS enzyme inactive [13,22]. MSP2 strain showed low expression of *glnA1* gene as compared to the expression in other strains in low nitrogen condition because there was no regulation at transcriptional level due to lack of P1 promoter hence lack of GlnR binding motif also.

PLG layer has been known to be present in the cell wall of only virulent strains of mycobacteria [16,23]. Harth and colleagues indicated that extracellular GS of pathogenic mycobacteria is involved in synthesis of this layer [10,24,25]. There has also been reports stating the involvement of PLG layer of *M. bovis* in cell wall strength and in providing resistance to various physical and chemical stress factors [8]. The absence of PLG layer from the cell wall of mycobacteria grown in high nitrogen condition indirectly suggest that PLG layer may be a form of nitrogen assimilation in pathogenic mycobacteria. In macrophages, mycobacteria encounter nitrogen stress which leads to high GS expression and PLG layer synthesis in the cell wall. Immunogold localization and PLG isolation studies further validated the finding of no detectable PLG in the cell wall of *M. bovis*, MSFP, MSP1 and MSP2 strains when grown in high nitrogen conditions.

The ability of the pathogenic mycobacteria to form biofilm adds on to their virulence potential [26]. Biofilm formed at air liquid interface are popularly known as pellicle. Additionally, mycolic acids are the major component of the biofilms formed by mycobacterial species [26,27] but it is not clearly known whether mycolic acid synthesis or its amount in cell wall is affected by PLG layer. However, there are few reports that suggest the involvement of PLG layer in biofilm formation [8]. A ∆*glnA1* strain of *M. bovis* that lack PLG layer in the cell wall was found to be defective in biofilm formation [8]. Additionally, our results showed that the biofilm and pellicle forming capability of *M. smegmatis* strain complemented with *M. bovis glnA1* was enhanced than the wild type. This is due to the fact that higher expression of *M. bovis glnA1* leads to the synthesis of PLG layer in the *M. smegmatis* complemented with *M. bovis glnA1*[8]. There are reports also suggesting that microbial amyloids play a significant role in biofilms of actinobacteria [28,29]. Additionally, it was observed that biofilm was formed significantly much better in low nitrogen conditions which added to the involvement of PLG layer in biofilm formation.

There is a gap in our understanding of the exact mechanisms and enzymes involved in the synthesis of PLG layer till date. In addition to it, characterization of PLG layer, can further help in our understanding of complex mycobacterial cell wall. Because of high molecular weight and inert nature of the polymer it may also act as an adjuvant. This needs further investigation. Establishment of the pathways involved in PLG synthesis will further help in identification of new drug targets against tuberculosis. The study of nitrogen metabolism can provide an insight in the survival of these pathogens in adverse conditions for long duration of time. Also this can help us to understand the mechanisms by which bacteria are able to survive and replicate in macrophages.

## Conclusions

In the current study we have investigated the expression of *glnA1* gene of *M. bovis* in response to nitrogen availability. This study revealed for the first time that amount of PLG in the cell wall of *M. bovis* is substantially reduced when grown in high nitrogen conditions. The data presented here significantly enhance our understanding of the regulation of the *glnA1* gene which is linked to synthesis of the PLG layer in the cell wall of *M. bovis* in altering nitrogen conditions. The localization study of PLG layer in the cell wall, as shown by immunogold studies has also been reported for the first time.

## Abbreviations

LN: Low nitrogen; HN: High nitrogen; PLG: Poly-L-glutamine; GS: Glutamine synthetase.

## Competing interests

The authors declare that they have no competing interest.

## Authors’ contributions

DT designed, performed and analyzed the experiments. DT and RB wrote the paper. RB contributed reagents, materials and analysis tools. HC made the MSP2 construct for this study. All authors have read and approved the manuscript.

## Supplementary Material

Additional file 1: Table S1 Primers used for cloning and real time PCR. Click here for file
