# Supplementary material for: Poly-L-glutamate/glutamine synthesis in the cell wall of Mycobacterium bovis is regulated in response to nitrogen availability
Source: BMC Microbiol. 2013 Oct 11;13:226. doi: 10.1186/1471-2180-13-226 (PMC3852478; doi:10.1186/1471-2180-13-226)
Supplement: Additional file 1: Table S1 — Primers used for cloning and real time PCR. [file 1471-2180-13-226-S1.docx]

**Additional file 1: Table S1.** Primers used for cloning and real time PCR

| No. | Oligonuleotides | Sequence (5’ → 3’) |
| --- | --- | --- |
| 1.  2.  3.  4.  5.  6.  7.  8.  9.  10.  11.  12. | Primer 1  Primer 2  Primer 3  Primer 4  Primer 5  Primer 6  Primer 7  Primer 8  Primer 9  Primer 10  Primer 11  Primer 12 | 5’ *GGA TCC* GCG ATC AGC CAG TCG ATC3’  5’ *CTG CAG* TTA AAC GTC GTA GTA CAG3’  5’ *GGA TCC* GGG CCG GGG AGA CCC GGC 3’  5’ ATC GGC TCC ATA GCG TGC 3’  5’ ACG TTA CTG ACC GTG CCG 3’  5’ GTT CCA GTC GAT CCA CGA AT 3’  5’ GTG CTG GCC AGG TAG TTC TC 3’  5’ CTC GGT TCG CGC CTA CCT C 3’  5’ CGT CGC GGC AGA TCC AC 3’  5’ TTC CAG TCG ATC CAC GAA TC 3’  5’ GTC GCG CAG GTC GAC3’  5’ GGG CCT GGT CGG CCA TGG CGC3’ |

Restriction sites mentioned in italics letter
